# Consumer Eating Behavior and Opinions about the Food Safety of Street Food in Poland

**DOI:** 10.3390/nu13020594

**Published:** 2021-02-11

**Authors:** Michał Wiatrowski, Ewa Czarniecka-Skubina, Joanna Trafiałek

**Affiliations:** Department of Food Gastronomy and Food Hygiene, Institute of Human Nutrition Sciences, Warsaw University of Life Sciences (WULS), str. Nowoursynowska 166, 02-787 Warsaw, Poland; michal_wiatrowski@sggw.edu.pl (M.W.); joanna_trafialek@sggw.edu.pl (J.T.)

**Keywords:** street food, consumer, customer behavior, food safety, Poles

## Abstract

Street food plays an increasingly important role in the nutrition of the inhabitants of European cities. Our study aimed to analyze Polish consumers’ attitudes toward food offered in street food outlets, consumers’ eating out behavior, and the factors that determine their choice of meals from street food vendors. A survey was conducted of 1300 adult respondents who eat street food in Poland. The research enabled a detailed and comprehensive assessment of consumer behavior toward the use of street food outlets, as well as consumer opinions on vendors’ functioning, including hygiene and meals offered. Factors determining the frequency of street food consumption, preferred food types, and factors influencing the use of such outlets were identified. The most important factors were the quality of services and meals, personal preferences and price. Using cluster analysis, consumer profiles based on the types of street food outlets and food preferred were identified. Four main street food consumer preference profiles were identified: ‘burger-enthusiasts’, ‘kebab-enthusiasts’ and ‘ice-cream enthusiasts’, and ‘no specific-oriented consumers’. The Internet and social media were identified as information and promotion channels for this form of gastronomy. Results also revealed Polish consumer behavior and opinions about the food safety of street food in Poland. In summary, in Poland the habits of eating typical Polish homemade dishes is being replaced by eating meals in street food outlets, which can be classified as fast food. Increasing consumer knowledge and awareness of the quality and safety of street food may counteract improper hygiene practices of sellers.

## 1. Introduction

Street food terminology defines food and beverages as either ready for direct consumption or not and includes food that is ready-to-eat and food that is minimally processed, prepared and/or sold by vendors and handlers. This is an inexpensive food for all workers of all socio-economic classes and occupations [1,2].

Street food plays an increasingly important role in the food intake of Europeans [3]. Street food has been historically popular especially in cities of third world and developing countries, for low and middle wage-earning classes of people [4,5,6,7]. This form of gastronomy, which has been popular in Asian and African countries and in Latin America for a long time, began to penetrate into Europe and North America with an influx of migrants and European and American tourist trips. It has become a channel of food novelty [8].

Currently, food-trucks are a restaurant marketing strategy in Brazil and in Paris [3,9]. Street food is an alternative to delivering food to or near places with high traffic, such as schools, parks, gardens, markets, avenues, office buildings, and tourist areas. It allows vendors to implement a unique menu while using simplified techniques for preparing meals and provides an alternative to homemade food. Many authors [10,11,12,13,14] have identified features of street food, including practicality, saving time in the preparation of dishes, convenience, seasonality, lack of restrictions, and low costs of entering the market, as well as relatively low meal prices for consumers. Moreover, they are quick alternatives to restaurants, even during late hours after other food establishments are closed. Street food usually represents local culture and traditions [10,11,15,16,17], and the menu is distinguished in each country by its regional specificity.

Due to their locations on the street, the hygienic conditions of preparing and selling food by street vendors are often unacceptable [16,17,18,19]. Studies about street food focusing on food safety and on vendors’ food safety knowledge and use of hygienic practices, as well as on the microbiological quality of street food products, are still limited to continents such as Africa [20,21,22], South America [23,24,25,26,27,28], central America [29], and Asia [30,31,32,33,34,35,36]. Studies conducted on European street food evaluated the hygiene of street food vendors and the microbiological quality of food products [3,28,31,32,37,38,39]. Because of this, current assessments of street vendors are largely based on data from developing countries. Many authors [29,40,41] have indicated that vendors are very often poor, uneducated, and unconcerned about the safe handling of foods, and vendors could provide neither proof of food safety training nor a valid license for street trading. Street foods have been implicated in outbreaks of foodborne illnesses, in particular Salmonella infections [16,33], as well as contamination with coliforms [34], bacterial contaminations, and other infections as a result of transmission from vendors’ [29,42,43,44].

Due to the poor hygienic conditions and the lack of vendor awareness, foodborne infections from street food occur in both developed and undeveloped countries [25,45]. This is associated with poor food safety knowledge and food safety attitudes, as well as poor hygienic practices of food vendors, which in many cases are associated with the lack of running water facilities, and exposure of utensils and foods to insects and dirt [22,26,27,29,36,46,47,48,49,50,51]. In a few cases, hygienic production in street food facilities is not properly established or monitored by national food sanitation groups, especially in developing countries [35,52,53]. Despite the food safety practices and requirements for street food in more advanced developed countries [54,55], food-borne illnesses and related risks are experiencing a rise [56]. This is due to globalization, and a wide variety of ethnic and local foods from throughout the world being sold by street food vendors [57,58,59]. Few publications exist referring to street food outlets and their specifications in European countries, especially in Poland, where street food has become popular in the last two decades. Therefore, further research is needed to examine the characteristics and safety of street food, as well as consumer behaviors related to street food in developed economies. Our study fills a research gap in the literature on this topic by investigating Polish customer opinion about street food, taking into consideration food safety, customer eating behavior and preferences, as well as factors determining the choice of street food.

Studies of street food establishments primarily focus on food choices and the frequency of using street food [5,9,11], determine the nutritional value and risk of developing diet-related diseases [12,25,52], assess the risk of health hazards to consumers [15,16,19,24,34,35,44,48,49,50,51]. Such information rarely reaches consumers and will not influence their behavior. Therefore, it is important to consider a quick, visual assessment of food production and distribution conditions not only by sanitary services, but also by consumers.

Consumers do not exert pressure on street vendors to improve food preparation conditions [60]. Sanitary inspections are insufficient for ensuring safety. Some authors [58] suggest that consumers should be aware of the risk of consuming street food. Many Greeks and Poles have been served low-quality meals and have made complaints [19]. According to Okumus and Sonmez [61], prior to consuming food, customers should carefully observe whether food truck operators have a license, check the personnel’s hygiene (such as clean hands, clean and short nails, gloves use, covered hair, beard, mustache, and appropriate clothing: apron, uniform), check the temperature of dishes (hot food hot, cold food cold), as well as check the service area for sanitary conditions (including clean table, clean service utensils and garbage container availability). If food is not prepared according to these ‘critical control points’, consumers should avoid consumption there.

Three decades ago in Poland, people usually ate homemade meals. Currently, the lifestyle and habits of consumers have changed and influenced foodservice sector development. Among other factors, global trends such as demographic changes (growth of 1–2 members households), economic changes (average household income growth), as well as consumers’ knowledge growth (on topics: food, nutrition, health) have all contributed to changes in consumer food consumption [62]

Over the two last decades, the amount of money in Polish households allocated to catering services has increased, and the share of food spending and total expenditure doubled, despite the amount being low. The average individual Polish spend on food out of home varies between 26.2 PLN (about 8$ in 2013) to 60.20 PLN (about 16.5$ in 2015), which accounted for 8.5% of their total food budget and 1.9–3.0% (2015–2020) of the total consumption expenditures. For 27 European Union (EU) countries the average share of consumption expenditure equals 7%. The share of household expenditure devoted to catering services was the largest in Ireland (14.4%), Spain (13.0%), Malta (12.6%) and Greece (12.4%), but the lowest in Poland (only 3% in 2017). This indicates a low preference of Poles to eat away from home [62,63,64,65].

Therefore, our research aimed to analyze consumer attitudes toward street food (kiosks, stalls, food trucks) and eating out behavior, as well as the factors determining the choice of meals in street food outlets.

The following research questions were formulated:

- What are the motives (factors) of consumers choosing street food outlets?
- What consumer profiles can be identified according to the frequency of using street food outlets?
- How do consumers evaluate street food outlets in terms of food, including food quality, service, and hygiene?

## 2. Materials and Methods

### 2.1. Data Collection

We designed a questionnaire based on available questionnaires [66,67] and our previous research related to street food [3]. The questionnaire was assessed by determining its repeatability. The reliability of the questionnaire was validated using its internal consistency. Cronbach’s alpha test was used to measure internal consistency and reliability. Cronbach alpha coefficient was above 0.7, which indicated acceptable internal consistency. Therefore, the questionnaire and scale used is valid. A pretest of the questionnaire was performed through a pilot study (*n* = 12) within the population of interest. This group was not added to the main research. All problems were identified, and the questionnaire was completed and corrected. The data were collected by the authors using the PAPI (pen-and-paper interview) method.

Inclusion criteria of respondents for main study were as follows:

1. Each respondent who agreed to participate in the survey was invited to complete the questionnaire. If necessary, explanations were provided.
2. Everyone, independent of age, using the offer of the street food did not suffer from diseases requiring a special menu offer.

The exclusion criterion of respondents was people who don’t use street food outlets. The participants in the study were a convenient sample of consumers. They were free to participate in the questionnaire.

The questionnaire consisted of two parts (Appendix A, Table A1). The first part of the questionnaire included nine questions, relating to consumer behavior in various street food facilities and consumer attitudes toward food offered in those places. Consumer behavior was analyzed based on the frequency of use of street food outlets, factors influencing the use of those facilities, choice of street food products, assessment of hygienic factors in the outlets, and customers opinion of the street food. The second part of the questionnaire was related to respondent sociodemographic details: gender, age, education, dwelling place, respondents self-reported financial status.

### 2.2. Data Analysis

The statistical analysis of the results was performed using Statistica software (version 13.3 PL; StatSoft Inc., Krakow, Poland). The analysis of variance (ANOVA) test and multi-dimensional cluster analysis were used. Significance of differences between the values was determined at a significance level of *p* < 0.05.

A multi-dimensional cluster analysis calculation- was performed: hierarchical cluster analysis. The goal of our cluster analysis calculation was to build a tree diagram where the answers given by participants were most similar in a specific cluster. In order to avoid high correlated variables distortions on results, the variance inflation factor (VIF) has been calculated for the possibility of using cluster analysis. For cluster validation statistics we used internal measures for cluster validation, e.g., the matrix and Euclidean distance. The Ward method, as a hierarchical clustering method, was used to create groups, where the variance within the groups is minimized. We used the cluster analysis to determine consumer profiles based on the reasons for using and not using street food outlets and their opinions about these. In the cluster analysis of consumer opinion profiles, features such as price, service, hygiene, promotion, organization, and quality were taken into account, as well as individual component variables.

## 3. Results

### 3.1. Characteristics of Respondents

The characteristics of the respondents are presented in Table 1; 1300 people, including 54.2% of women, took part in the study. The study included young females and men between the age range of 19 and 30 years (70.2%) with secondary (52%) and higher education (39.5%), living in cities. Participants mainly reported ‘very good’ and ‘good’ financial status (60.2%). A smaller percentage of the respondents declared their financial status as ‘not good, not bad’ (29.6%), and as ‘bad’ (10.2%). Street food was used by all respondents.

### 3.2. Use of Street Food Outlets by Polish Consumers

Many respondents (*n* = 1131, 87%) regularly used street food services. The others (*n* = 169, 13%) used this form of gastronomy occasionally, e.g., at street food festivals, outdoor events, etc. A statistically significant influence of gender (*p* = 0.00076), age (*p* = 0.00006), education (*p* = 0.0031), dwelling place (*p* = 0.00016), and financial status (*p* = 0.00001) on using street food outlets was found. This form of gastronomy is significantly more often used by men, people aged 19–30 years, with lower than secondary education, living in the countryside or in cities up to 250,000 of residents, and a ‘bad’ financial status.

The respondents visited street food outlets with varying frequency. This form of catering was used by a small percentage of consumers every day and four or five times a week (*n* = 73, 5.6% and *n* = 179, 13.8%, respectively). The highest percentage of respondents visited two or three times a week (*n* = 309, 23.8%) and once a week (*n* = 216, 16.6%). The remaining respondents chose these facilities less frequently: once a month (*n* = 182, 14%), once every 2 or 3 months (*n* = 213, 16.4%) or less often (*n* = 128, 9.9%). The frequency of using this form of gastronomy depended on gender (*p* = 0.00011), age (*p* = 0.000001), education (*p* = 0.00014), dwelling place (*p* = 0.00006) and financial status (*p* = 0.000001). Men, and people under the age of 18 years, with the lowest education, living in the countryside or in small towns, and with a ’bad’ financial status used this form of eating outside the home significantly more often, visiting this type of facility every day or several times a week. It is probably the cheapest form of dining away for them when they are at school or at work.

The choice of street food depended on gender (*p* = 0.0010), age (*p* = 0.000001), education (*p* = 0.000001), financial situation (*p* = 0.00001), and dwelling place (*p* = 0.0014).

**Figure 1:**
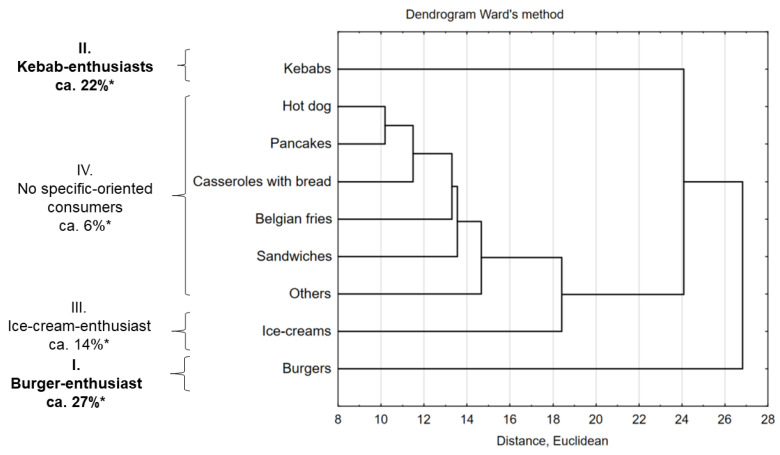
Consumers’ street food profile taking into consideration meals (* percentage of responses; others—Asian, Italian, Tex-Mex meals or just places with good food).

Based on the cluster analysis, the profiles of preferences of consumers using street food were determined (Figure 1). Four profiles were identified:

- burger-enthusiasts (I), young consumers (aged 19–30 years), mainly men, highly educated, with ‘good’ and ‘very good’ financial status (26.6%, *p* = 0.05) who used street food for three or four times a week;
- kebab-enthusiasts (II), young respondents (aged 19–30 years), mainly men (22.6%, *p* = 0.05), who used street food for three or four times a week;
- ice-cream enthusiasts (III), consumers with various sociodemographic groups (13.3%, *p* = 0.05), who used street food once a month;
- no specific-oriented consumers (IV), respondents, mainly women, secondary educated, who used street food with different frequency.

Consumers consumed burgers (*n* = 346, 27%), kebabs (*n* = 294, 22%) and ice cream (*n* = 173, 14%) most often. Other consumer profiles were represented by small groups of respondents (between *n* = 39 to *n* = −99, between 3% to 8%).

### 3.3. Reasons for Using Out-of-Home Eating and Choosing Catering Establishments

Among the reasons for eating outside the home, respondents mentioned social gatherings, convenience, reluctance to prepare meals oneself, and discovering new flavors (Table 2).

The respondents use various sources when choosing a catering establishment. Most often these were the opinions of friends, family (*n* = 425, 32.69%), or social networking sites and internet forums (*n* = 399, 30.69%). They also used websites with restaurant reviews (*n* = 180, 13.85%), articles on the Internet (*n* = 137, 10.54%), articles in the local press and in weeklies (*n* = 47, 3.62%), blogs (*n* = 72, 5.54%), and others (*n* = 40, 3.08%). The following other sources were listed: TV and advertisements such as leaflets, folders, phone applications, radio, advertising on the roads, vlogs, Google Maps, and the website streetfoodpolska.pl. The choice of the source of information about catering establishments depended on gender (*p* = 0.00001), age (*p* = 0.000001), education (*p* = 0.000001), dwelling place (*p* = 0.0001), and financial situation (*p* = 0.000001).

**Figure 2:**
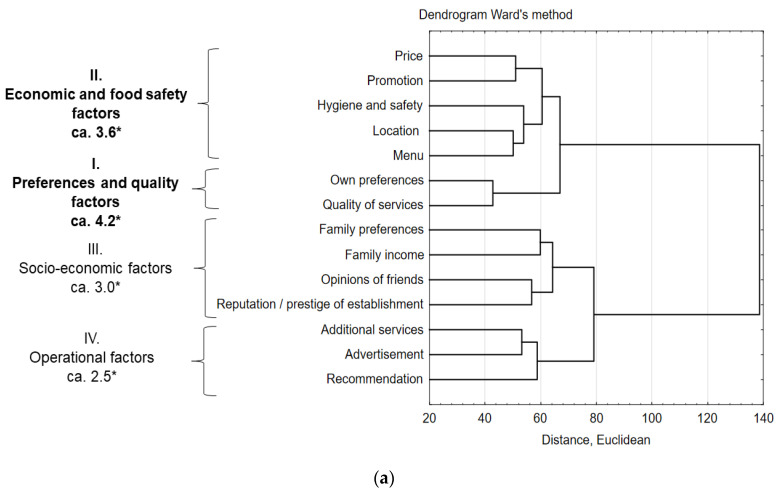

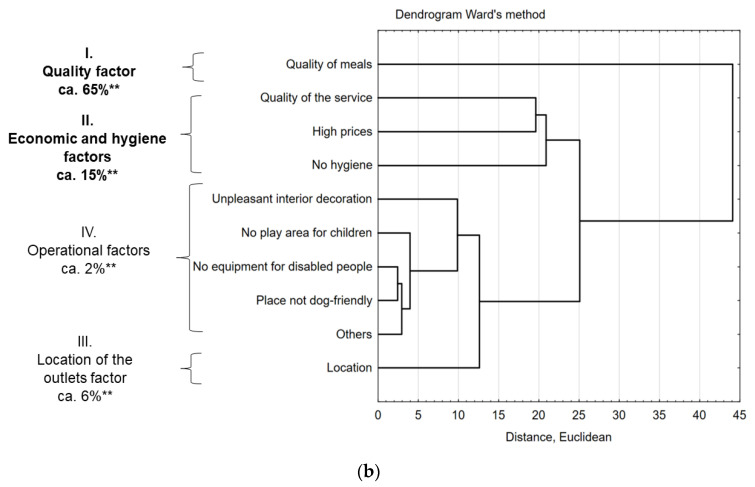
Factors determining (**a**) and discouraging (**b**) the selection of street food outlets (* factor importance on a 5-point scale, ** consumer % answer on the factor importance).

Based on the results and cluster analysis, the factors indicated by the respondents were grouped into factors that determine (Figure 2a) and discourage consumers (Figure 2b) from choosing catering establishments. Factors from both groups were divided into four clusters of factors, of which factors in groups I and II were high or medium decision power, while factors from groups III and IV had a little influence on consumer decisions. The choice of a catering establishment was determined by the following factors: I—preferences and quality, II—economic and food safety, III—socio-economic, and IV—operational. Among discouraging factors for visiting catering establishments, the following were distinguished: I—quality factor, II—economic and hygiene factors, III—location of premises factor, and IV—operational factors.

The most important factor determining the selection of establishments was the preferences and quality factor, which took into account the quality of services and personal preferences (score 4.2 on a 5-point scale). Similarly, the most important discouraging factor was the quality factor. It was indicated by over 65% of consumers. Other components of individual factors in both groups are presented in Figure 2a,b. The figure also shows the results of the scoring scale calculations for determining factors and the percentage of consumer responses when disincentives are present.

The presence of the ‘preferences and quality’ factor in decisions for choosing a catering establishment is confirmed by the calculations of descriptive statistics, i.e., mean, SD, and medians. In the case of the expectation of the quality of service and the satisfaction of an individual’s own preferences (factor I, Figure 2a), the obtained mean was the highest and the median equal to the highest value of the scale (mean = 4.20, SD = 1.09, median = 5.0; and mean = 4.19, SD = 1.13, median = 5.0, respectively).

As a discouraging factor, over 65% of consumers (SD = 4.75) have indicated ‘quality’ (factor I, Figure 2b). The factor moderately influencing the choice of premises was the ‘economic and food safety’ factor (factor II, Figure 2a). The mean of the components of this factor was smaller and ranged between 3.50–3.71 (median 4.0). High price, along with the lack of hygiene and the quality of service, was a factor that moderately discouraged people from visiting the premises again (mean 15.5% of responses, SD = 3.8), and is labelled ‘economic and hygiene’ (factor II, Figure 2b).

The other factors, i.e., ‘socio-economic’ and ‘operational’ (factor III and IV, Figure 2a), had a slight influence on consumer decisions (mean = 2.88, SD = 1.23, medians 2–3). Similarly, factors of social significance, i.e., ‘location of the outlets’ and ‘operational factors’ (factor III and IV, Figure 2b), were not-important in decisions to re-visit the premises (mean 4.1% of responses, SD = 3.0).

### 3.4. Consumer Opinion about Street Food Outlets

When asked for their opinion on street food establishments, the respondents agreed that this is a new type of cuisine which is becoming more and more popular (median 4, Table 3). They perceive these establishments as different than fast food, but in their opinion, it is neither a healthier version of fast food, nor a cheaper offering (median 3, Table 3). According to them, this type of catering establishment has good hygiene and food quality similar to typical catering establishments. However, the consumer opinions presented in our study, in most of the responses was between ‘undecided’ and ‘moderately agree’. Respondents did not agree with the following statements: ‘unnecessary outlets that worsen the image of the city’; ‘facilities with a low hygiene level’ and that ‘food quality worse than in typical (non-street) catering establishments’. It should be mentioned that statements used in our questionnaire were based on the literature, consumer opinions presented on the Internet, and on preliminary research.

The respondents (*n* = 397, 30.5%) sometimes complained about the quality of the dishes. A small group of respondents (*n* = 148, 11.4%) very often reported complaints about catering services. Other respondents almost never (*n* = 483, 37.2%) and never (*n* = 272, 20.9%) made any complaint about the quality of the dishes. Complaint about food quality depended on the age (*p* = 0.00001), education (*p* = 0.000001), dwelling place (*p* = 0.00181), and financial situation (*p* = 0.000001) of the respondents. People aged 31–55 years, with vocational and elementary education, living in cities of up to 250,000 inhabitants, and with ’bad’ financial situations made complaints significantly more often.

Consumers’ opinion on hygiene in street food outlets was examined (Table 4). The questions inquired about the necessary hygiene requirements in food production and the requirements specified in the *Codex Alimentarius* [68].

Although the respondents were not experienced hygiene auditors, based on their observations regarding the recently visited street food outlet, they indicated irregularities in the field of hygiene, specifically in the areas of personal hygiene of staff, hygiene conditions of food production, as well as hygiene of food production and distribution (Table 4).

In the respondents’ opinion, employees of street food outlets did not protect hands from injuries (79.5% responses). They wore jewelry on their hands during work (51%), and they had no or did not change disposable gloves frequently enough (41.5%). They touched their face, hair, nose, or ears during food production (40%); had no protection from their long hair (32.2%) while working with food; had inadequate working clothes (30.5%); and the payment process was not properly separated from food production (28.7%). Based on the observations of the respondents, it can be said that the personal hygiene of street vendors was not fully compliant with hygiene standards.

The respondents noticed irregularities in the hygiene conditions of food production as follows: overflowing waste bin in the production area (86.7% responses) and the presence of employees’ personal items (phones, bags) in the production area (44.7%).

In the area of hygiene of food production and distribution processes, the respondents noticed that ready-to-eat products and wasted ones were not separately stored (79.2%), and they observed unauthorized people in the production areas (38.9%).

## 4. Discussion

### 4.1. Use of Street Food Outlets by Polish Consumers

Culinary preferences and eating habits have changed over time and are inextricably linked with human history. However, globalization is accelerating the pace of these changes more than in previous centuries. Economic progress, development of food techniques, and technology have brought many benefits, among them the speed of work and rest as well as the speed and convenience of obtaining and preparing a meal. On the other hand, living in a hurry, eating ‘ad hoc’ food or fast food, often non-compliant with the nutritionists’ recommendations, and diet-related diseases are a consequence of cultural change. Changes in eating habits occur not only between generations, but also among those who change their current model of nutrition to a new one in a relatively short time. The main causes of change in dietary preferences are factors such as migration, new ways of processing and storing food, international trade development, increases in levels of wealth, changes in family functioning, an increase in environmental awareness, fashion, etc. [8].

Globalization causes the culinary traditions to be transferred from one place (region, country) to another. However, it can be a kind of threat, leading to the universalization of eating habits and the disappearance of local culinary traditions [70]. Poland is such a case, where the habits of eating typical Polish homemade dishes are replaced by gastronomy meals, which are very often eaten in fast-food establishments.

The interest in, and increased use of, catering services in Poland is due not only to having insufficient time to prepare meals, but also individuals lacking skills to prepare them, greater women’s involvement in professional work and returning home late with too many professional duties outside the home, an increase of 1–2 person households, as well as the increased income of Poles [71].

Catering establishments take advantage of consumers’ interest in eating out and adjust their offerings to various social groups and their expectations and financial capacity. The constant rush and lack of time to eat and prepare a meal, as well as the relatively high cost of meals in traditional gastronomy, are the factors contributing to street gastronomy gaining popularity in Poland. Because it is mobile, it can reach consumers directly and offer simple dishes close to workplaces, tourist attractions, and other frequently visited places. All respondents participating in our research used street food services, 87% of which did so regularly.

This is a result of the growing popularity of street food not only in Poland but all over Europe [3]. In Poland, the growth of street food outlets is fostered by various types of outdoor, tourist and cultural and entertainment events, as well as sports and recreation events, which attract customers and usually take place in places without permanent catering establishments [8,72,73,74,75,76,77].

In the group of consumers assessed, the choice of burgers and kebabs dominated, having become very popular. This is one example of the transfer of eating habits from one culture to another. In Poland, street food, especially offered in food trucks, plays an important role in promoting the cuisine of other countries, often for exotic-craving Poles. The average Polish consumer, when dealing with street food dishes, has the opportunity to learn new tastes, which may have an impact on changing current culinary preferences.

Owners of street food outlets develop their own gastronomic activity based on their impression of and fascination with other cultures and cuisines. For example, ‘Carnitas Food Truck’ (in Warsaw) specializes in street food typical for Mexican cuisine such as tacos, burritos with salsas chili habanero, chipotle, and guacamole. The food truck ‘La Chica Sandwicheria’ (in Warsaw) specializes in food typical of Cuban cuisine. Sometimes globalization of cuisine is seen in examples such as the food truck ‘Pepe Crepe’ (in Warsaw) that offered Japanese-style crepes that are not traditional but contemporary, globalized versions of crepes. Street food is offered at many culinary festivals in Poland, such as Slow Weekend and Asian Street Food Fest [8]. As research [8] indicates, the majority of people visiting street food festivals are people aged 20–30, sometimes with small children.

Respondents used street food usually two or three times a week and once a week. Our results are similar to the results of Kowalczuk [78], who has shown in her research that an average Pole (*n* = 1013) visits foodservice outlets once a month, with the individuals being slightly more often men and far more often young people, and those with middle and higher income. People with secondary and higher education benefit more commonly from food services, including school and university students and white-collar workers, who live in large cities. It should be mentioned that participants in our study were mainly people under 30 years of age (about 85%), with secondary and higher education (91.5%), and who lived in a big city (about 70%).

Based on the analysis of clusters due to the choice of menu, preferences of street food consumers were identified in our study. They are: ‘burger-enthusiasts’, ‘kebab-enthusiasts’, ‘ice-cream enthusiasts’, and ‘non-specified-oriented consumers’. Levytska and Kwiatkowska [79] have indicated that Polish consumers are changing preferences for foodservice outlets (from fast-food outlets to casual dining restaurants and quick service restaurants—QSR), and change preferences for menu offerings. According to those authors, Poles have begun to choose foods with reduced energy value (light), foods with a modified composition of nutrients and functional foods (with proven, beneficial effects on health), organic food, and conventionally produced food. But as stated in our study, consumers of street food preferred fast food products like burgers, kebabs, ice-creams, and occasionally other food like Asian, Italian, and Tex-Mex cuisines. Very similar results were obtained by Kolanowski et al. [19] among Polish and Greek consumers. Greeks preferred pizza and creperies while Poles preferred pizza and kebab. This is in line with the characteristics presented by Kowalczuk [78], who divided modern Polish consumers into three groups by interests: ‘health and safety’, ‘convenience’, ‘pleasure and experience-seeking’. Polish and Italian cuisines are types of cuisine and dishes that Polish consumers preferred. Younger respondents are also open to dishes from other countries (Chinese, Greek, Turkish, Japanese). Meat dishes and hot snacks like burgers and kebabs are the most preferred, cold snacks and vegetarian dishes are the least popular [62,74,75,76].

### 4.2. Reasons for Using Out-of-Home Eating and Choosing Catering Establishments

Street food consumers mentioned the following main reasons for eating outside the home: getting to know new tastes, socializing, convenience and lack of time, celebrating special occasions, and reluctance to prepare dishes on their own. Other authors have also pointed out reasons such as convenience and saving time [62].

The main reasons for consumers using catering outlets are the taste of dishes, reasonable prices, convenience, and saving time, while the main barrier is a lack of money. Consumers tend to spend more money in catering outlets per month than in the past [62], and the amount largely depends on the income level, but also age, education, and place of residence. The reason for the occasional use of catering services is also based on the common Polish belief that home-cooked meals are healthier.

Polish respondents use a variety of sources when choosing a catering establishment, including street food outlets. Most often these are the opinions of friends and family, social networks and Internet forums, websites with restaurant reviews, articles on the Internet and articles in the press, blogs, and others. This is a typical behavior in the era of globalization. It has been observed that the following mass media sources play an important role in promoting catering services and influencing culinary tastes in Poland: daily press, weeklies and monthly magazines, television (travel programs available in Poland: ‘Travel Channel’, ‘National Geographic Channel’, ‘Planete+’), and especially the Internet (social networks, blogs, etc.) [8]. According to market reports [76,80] the main sources of information about food services are friends and family, while half of the respondents have also pointed to the Internet, including social media.

Among the factors in our study determining the selection of catering establishments, the most important role was played by ‘preferences and quality’ and ‘economic and food safety’. The most important discouraging factors were ‘quality’ and ‘economic and hygiene factors’.

According to market reports [76,81], respondents considered unpalatable food as the most discouraging factor from visiting establishments again. Another reason was the lack of hygiene in the outlets. Other disincentives were shown by Kolanowski et al. [19] and differed depending on the country of the study, i.e., in Poland, it was a strange taste of dishes, poor services, and lack of hygiene, and in Greece, lack of cleanliness and price. The results of our research confirm previous research and allow us to conclude that consumers are paying more attention to the quality of prepared meals and the hygienic condition of catering establishments.

### 4.3. Consumer Opinion about Street Food Outlets

The form of street food in Poland is typical in developed countries [82]. Despite the fact that results of various studies [73,74,75,76,77] have indicated that Polish consumers prefer typically Polish dishes, in our study in street food outlets, they mainly order burgers and kebabs, which are classified as fast food, as well as ice cream as a dessert. It should be emphasized, however, that the products offered are often original dishes, prepared according to proprietary recipes by the owners or employees of the street food outlets. These are high-energy products and are not recommended by dietitians. The Makro Cash and Carry market report [75] shows that the most popular dishes among Polish street food offerings are kebabs (50%, *n* = 1000), pancakes (32%), casseroles with bread (31%), burgers (30%, *n* = 1000), and Belgian fries (11%, *n* = 1000).

Various authors have tried to characterize the most popular dishes found in mobile gastronomy in developing countries [83] and found them to be typical, local, and very diverse dishes, derived from the regions in which they are sold. Other authors [84,85] have described the most popular meals sold by street food vendors in Europe. They identified pancakes in France and Italy, sausage and yeast dough in the Czech Republic, and chicken dishes, pancakes with meat, and quail eggs in Spain.

These results are similar to those of other authors. Different studies state that street food meals are characterized by low nutritional value and contain large amounts of carbohydrates and fats, especially saturated fats [86], and street sweets and sweet beverages are products with a high levels of ingredients like sugar, saturated fat, trans-fatty acids, and salt [83,87,88].

Moreover, many studies [83,89,90,91,92] have shown that despite good nutritional behavior, consumers using street food services had lower dietary diversity than consumers using fast food. In studies by Buscemi et al. [37] conducted in Palermo (Italy), it was found that people who use street food more often have a significantly higher body mass index (BMI), waist circumference, cholesterol level, and serum uric acid levels than consumers who use this type of gastronomy less frequently.

According to Nonato et al. [86], street food gastronomy can be a source of food safety problems, contributing to the development of food-borne diseases and chronic diet-related diseases, especially among consumers who frequently use street food services. However, according to Kolanowski et al. [19], it may be a source of physical hazard, like other food products [93].

Most respondents have a positive opinion of street food outlets. Consumers do not consider them to be a cheap way of eating meals and do not agree with the statement that these are premises with a low level of hygiene. They believe that such meals are as safe as those offered in stationary premises.

Although the greatest problems and the greatest negligence related to ensuring food safety occur in developing countries, this does not mean that in Europe and the U.S. [31,32,38,39,94] that the handling of food is flawlessly safe. In a study by Trafialek et al. [38], attention was drawn to the fact that establishments specializing in street food gastronomy do not have clean running water, have limited space for proper food storage, have employees who mishandle waste generated during technological processes, and lack proper staff hygiene.

In street food outlets, meals are prepared in the presence of consumers. It is worth disseminating the results of similar studies among a wide audience to increase food safety awareness among consumers. Consumers are able to enforce proper food safety and should react to non-compliance with hygiene rules. The situation reported in Ghana is not recommended [95]. Despite knowing the health risks of eating contaminated street food, local consumers eagerly used food street services and did not expect an improvement in food hygiene production.

#### Limitations

This study has some limitations in terms of both its methodology and its applicability. The sample selected for the study consisted mainly of young adults between 19 and 30 years; therefore, caution should be exercised in attempting to generalize the results to an entire population. In addition, the study was conducted only in large cities in Poland. Consumers’ perceptions and behavior may be different in other places. Another limitation is that the consumers of street food were from only one country. Despite the limitations, the results obtained are of practical importance, especially for food safety authorities and street food vendors and owners.

## 5. Conclusions

The forms of street food in Poland are forms typical in other developed countries, as evidenced by the identified consumer profiles. About half of the respondents were ‘kebab- and burger-enthusiasts’. Similar factors affect the behavior of Polish consumers regarding the choice of street food outlets and the lack of willingness to re-use this form of meal outside the home. Consumers’ preferences, pro-quality factors, such as quality of services, quality of meals, hygiene, and food safety, and economic factors, such as the price of a meal, are of primary importance.

The opinion of the surveyed consumers about food street outlets was not always positive. Consumers commented on many aspects of sanitary conditions of street food facilities, such as personal hygiene, hygiene conditions of food production, as well as hygiene of food production and distribution. For these reasons, the presented research is of practical importance for managers of these facilities and official quality control.

The results of the study can also be helpful for street food entrepreneurs because of the indicated identified promotion channels. It has been shown that the Internet is an important source of information on catering establishments, and social networking sites play a large role in consumer traffic and can be used for promotional purposes, practically without generating costs.

The quality of dishes, the quality of service, and hygiene are the factors that make consumers use a catering establishment. Constantly increasing consumer awareness in terms of proper nutrition and proper hygiene of food production may contribute to the improvement of quality in street food outlets.

Good sources that can encourage hygienic awareness by consumers come primarily from mass media, including the Internet and the website of sanitary inspections. Consumers should be made aware that proper food quality is both an appropriate nutritional value related to good manufacturing practice (GMP) and a result of food safety related to good hygiene practice (GHP). Consumer knowledge regarding what they should take into consideration when buying street food can be more effective than control and can better counteract improper hygiene practices of vendors.

The solution could be to create a street food digital application in which consumers could find information about street food and its nutritional value, but also could share their opinions on offerings, including their quality and observed production hygiene.

## Figures and Tables

**Table 1:** Characteristic of respondents.

Features of Population	Group	Number of Respondents (n)	Percentage of Respondents (%)
Total Gender	-	1300	100.0
	women	705	54.2
	men	595	45.8
Age	up to 18 years old	190	14.6
	19–30 years old	912	70.2
	31–55 years old	198	15.2
Education	vocational and elementary school	110	8.5
	secondary school	676	52.0
	higher education (university)	514	39.5
Dwelling place	city over 250,000 inhabitants	620	47.7
	city up to 250,000 inhabitants	291	22.4
	city up to 50,000 inhabitants	211	16.2
	village	178	13.7
Self-reported financial status	‘very good’	197	15.2
	‘good’	585	45.0
	‘not good not bad’	385	29.6
	‘bad’	133	10.2

**Table 2:** Reasons for using out-of-home eating.

Reasons	Average ± SD	Q25	Median	Q75
I like to meet my friends	3.71 ± 1.28	3	4	5
It is convenient	3.69 ± 1.15	3	4	5
I don’t have time to prepare meals myself	3.10 ± 1.33	2	3	4
I want to celebrate special occasions,	3.45 ± 1.36	2	4	5
I like to discover new flavors	3.72 ± 1.27	3	4	5
I don’t feel like cooking, I can’t cook,	3.05 ± 1.40	2	3	4
It is due to work (e.g., business meetings)	2.53 ± 1.37	1	2	4

Scale: 1—definitely do not agree, 2—moderately do not agree, 3—undecided; 4—moderately agree, 5—definitely agree; SD—standard deviation.

**Table 3:** Consumer opinion about street food outlets.

Opinion about Street Food	Average ± SD	Q25	Median	Q75
1: A new type of cuisine that is gaining popularity	3.41 ± 1.21	3	4	4
2: Another name for fast food	2.97 ± 1.29	2	3	4
3: A better and healthier version of fast food	2.96 ± 1.24	2	3	4
4: The cuisine designed for young people	2.84 ± 1.30	2	3	4
5: An element of the city’s landscape that enhances its image	2.67 ± 1.29	2	3	4
6: An unnecessary outlet that worsens the image of the city	2.27 ± 1.27	1	2	3
7: A way to attract more tourists to the city	2.93 ± 1.26	2	3	4
8: Cheap food	3.23 ± 1.20	2	3	4
9: Local cuisine	2.72 ± 1.22	2	3	4
10: An outlet wit facilities that have a low hygiene level	2.54 ± 1.18	2	2	3
11: Food with worse quality than typical (non-street) catering establishments	2.43 ± 1.05	2	2	3
12: Food with better quality than typical (non-street) catering establishments	2.87 ± 0.80	2	3	3
13: Food that quality is similar to typical (non-street) catering establishments	3.06 ± 1.12	2	3	4

1: definitely do not agree; 2: moderately do not agree; 3: undecided; 4: moderately agree; 5: definitely agree.

**Table 4:** The opinion of consumers about the sanitary conditions of street food facilities.

Opinion of Consumers *	Percentage (%)	
	Yes	No
Q.9.1. Is the production area of the facilities hygienic?	82.15	17.85
Q.9.2. Is there a waste bin available to employees in the production area and is it overflowing?	86.69	13.31
Q.9.3. Are the floors and facility walls in good condition (clean, undamaged, made from a smooth, easy to wash and disinfect material)?	78.62	21.38
Q.9.4. Are the production tops in good condition (clean, undamaged, made from a smooth, easy to wash and disinfect material)?	78.08	21.82
Q.9.5. Are there any food pests (rodents, insects) in the production area?	22.92	77.08
Q.9.6. Are there any personal items (phones, bags) of employees in the production area?	44.69	55.31
Q.9.7. Are raw materials stored in proper conditions (e.g., cold temperature)?	79.00	21.00
Q.9.8. Are ready-to-eat products and waste stored separately?	20.77	79.23
Q.9.9. Are catering tools clean and in a good condition (visually determined)?	80.62	19.38
Q.9.10. Are there any unauthorized people in the production areas?	37.38	62.62
Q.9.11. Do the raw materials look fresh?	82.54	17.46
Q.9.12. Do workers handle packaging hygienically?	75.77	24.23
Q.9.13. Do staff have clean hands during work?	81.85	18.15
Q.9.14. Are the hands of any employee with injuries protected?	20.54	79.46
Q.9.15. Do staff wear jewelry during work?	51.00	49.00
Q.9.16. Do staff have appropriate working clothes?	69.54	30.46
Q.9.17. Do staff protect their long hair (thus reducing the risk of food contamination)?	67.85	32.15
Q.9.18. Do staff wash their hands properly and frequently (by observation)?	74.77	25.23
Q.9.19. Is the payment process properly separated from production (e.g., by a different person accepting payment or covering of hands for hygienic tasks)?	71.31	28.69
Q.9.20. Do staff wear and change disposable gloves frequently enough?	58.54	41.46
Q.9.21. Do any staff have an illness (coughing, sneezing) that makes hygienic work difficult?	17.69	82.31
Q.9.22. Do staff touch their face, hair, nose, or ears during food production?	40.08	59.92

* References to criteria of assessment regarding Regulation (EC) 852/2004 [69].

## Data Availability

The data presented in this article is available on reasonable request from the corresponding author.

## Appendix A

**Table A1:** The questionnaire used in the study.

Question That Was Asked	Variants of Answers
**Q1.** How often do you use street food facilities? Choose the answers that suits you the best (only one option)	1: Every day; 2: Three or four times a week; 3: Once a week; 4: Two or three times a month; 5: Once a month; 6: Once every two or three months; 7: Less than once every two or three months; 8: Never (if respondents choose this answer, they end the questionnaire)
**Q2.** Factors (14) that you take into account when choosing a catering establishment: 1: Price, 2: Own preferences, 3: Family preferences, 4: Opinions of friends, 5: Additional services, 6: The reputation/ prestige of establishment, 7: Family income, 8: Recommendation, 9: Hygiene and safety, 10: Location, 11: Menu, 12: Quality of services, 13: Promotion, 14: Advertisement.	Choose the degree of importance for each factor (5-point scale). 1: Unimportant 2: Moderately unimportant 3: Neutral 4: Moderately unimportant 5: Very important
**Q3.** What are you doing when you’re looking for a new catering establishment? Choose the answers that suits you the best (only one option)	1: Ask your friends/ family; 2: Read articles on websites; 3: Check social media; 4: Check the establishment’s website; 5: Check websites with reviews of premises; 6: Read articles in the press; 7: Read internet blogs; 8: Others
**Q4.** Please comment on the following 7 statements about eating out: 1: I like to meet my friends, 2: It is convenient, 3: I don’t have time to prepare meals myself, 4: I want to celebrate special occasions, 5: I like to discover new flavors, 6: I don’t feel like cooking, I can’t cook, 7: It is due to work (e.g., business meetings).	Choose a comment for each statement (5-point scale): 1: Definitely do not agree; 2: Moderately do not agree; 3: Undecided; 4: Moderately agree; 5: Definitely agree
**Q5.** What discourages you from visiting an establishment again? Choose the answers that suits you the best (max. two option).	1: Quality of meals; 2: Quality of service; 3: High prices; 4: Unpleasant interior decoration, 5: Location; 6: No play area for children; 7: No equipment for disabled people; 8: Place not dog-friendly; 9: Lack of hygiene; 10: Others
**Q6.** Have you ever complained about the service in the catering establishments? Choose the answers that suits you the best (only one option)	1: Yes, very often; 2: Yes: sometimes; 3: Almost never; 4: No, never
**Q7.** Which street food outlets are your most favorite and/or most visited. Choose the answers that suits you the best (only one option).	Street food outlets serving: 1: Kebabs; 2: Burgers; 3: Hot dogs; 4: Belgian fries; 5: Casseroles with bread; 6: Pancakes; 7: Sandwiches; 8: Ice-Cream; 9: Others
**Q8.** Please comment on the following 13 statements: “Street food is: 1: A new type of cuisine that is gaining popularity”, 2: Another name for fast food”, 3: A better and healthier version of fast food”, 4: The cuisine designed for young people”, 5: An element of the city’s landscape that enhances its image”, 6: An unnecessary outlet that worsens the image of the city”, 7: A way to attract more tourists to the city”, 8: Cheap food”, 9: Local cuisine”, 10: An outlet with facilities that have a low hygiene level”, 11: Food with worse quality than typical (non-street) catering establishments”, 12: Food with better quality than typical (non-street) catering establishments”, 13: Food that quality is similar to typical (non-street) catering establishments”.	Choose a comment for each statement (5-point scale): 1: Definitely do not agree; 2: Moderately do not agree; 3: Undecided; 4: Moderately agree; 5: Definitely agree;
**Q9.** Observations concerning a recently visited street food premise, as well as 22 questions about hygiene in those facilities:	Choose the answers that suits you the best: Yes No
Q.9.1. Is the production area of the facilities hygienic?	
Q.9.2. Is there a waste bin available to employees in the production area and is it overflowing?	
Q.9.3. Are the floors and facility walls in good condition (clean, undamaged, made from a smooth, easy to wash and disinfect material)?	
Q.9.4. Are the production tops in good condition (clean, undamaged, made from a smooth, easy to wash and disinfect material)?	
Q.9.5. Are there any food pests (rodents, insects) in the production area?	
Q.9.6. Are there any personal items (phones, bags) of employees in the production area?	
Q.9.7. Are raw materials stored in proper condition (e.g., cold temperature)?	
Q.9.8. Are ready-to-eat products and waste stored separately?	
Q.9.9. Are catering tools clean and in a good condition (visually determined)?	
Q.9.10. Are there any unauthorized people in the production areas?	
Q.9.11. Do the raw materials look fresh?	
Q.9.12. Do workers handle packaging hygienically?	
Q.9.13. Do staff have clean hands during work?	
Q.9.14. Are the hands of any employee with injuries protected?	
Q.9.15. Do staff wear jewelry during work?	
Q.9.16. Do staff have appropriate working clothes?	
Q.9.17. Do staff protect their long hair (thus reducing the risk of food contamination)?	
Q.9.18. Do staff wash their hands properly and frequently (by observation)?	
Q.9.19. Is the payment process properly separated from production (e.g., by a different person accepting payment or covering of hands for hygienic tasks)?	
Q.9.20. Do staff wear and change disposable gloves frequently enough?	
Q.9.21. Do any staff have an illness (coughing, sneezing) that makes hygienic work difficult?	
Q.9.22. Do staff touch their face, hair, nose, or ears during food production?	
